# Thyroid hormone replacement one day before ^131^I therapy in patients with well-differentiated thyroid cancer

**DOI:** 10.7508/aojnmb.2013.01.005

**Published:** 2013

**Authors:** Daiki Kayano, Junichi Taki, Anri Inaki, Hiroshi Wakabayashi, Ayane Nakamura, Makoto Fukuoka, Seigo Kinuya

**Affiliations:** 1 Daiki Kayano: Department of Nuclear Medicine, Kanazawa University Hospital, kayano@nmd.m.kanazawa-u.ac.jp; 2 Junichi Taki: Department of Nuclear Medicine, Kanazawa University Hospital, taki@med.kanazawa-u.ac.jp; 3 Anri Inaki: Department of Nuclear Medicine, Kanazawa University Hospital, henri@nmd.m.kanazawa-u.ac.jp; 4 Hiroshi Wakabayashi: Department of Nuclear Medicine, Kanazawa University Hospital, wakabayashi@nmd.m.kanazawa-u.ac.jp; 5 Ayane Nakamura: Department of Nuclear Medicine, Kanazawa University Hospital, ayane@nmd.m.kanazawa-u.ac.jp; 6 Makoto Fukuoka: Department of Nuclear Medicine, Kanazawa University Hospital, fukuoka@nmd.m.kanazawa-u.ac.jp; 7 Seigo Kinuya: Department of Nuclear Medicine, Kanazawa University Hospital, kinuya@med.kanazawa-u.ac.jp

**Keywords:** thyroid cancer, ^131^I, hormone, replacement

## Abstract

**Objective:**

The current study aimed to determine the efficacy of radioiodine-131 (^131^I) ablation therapy with thyroid hormone replacement one day before ^131^I administration in patients with well-differentiated thyroid cancer (DTC).

**Methods:**

This retrospective study included 29 patients who underwent ^131^I therapies twice for DTC during 6-12 months. Since all the patients obviously had residual lesions by their serum thyroglobulin levels or their scintigrams at the first therapies, they underwent the second ^131^I therapies without diagnostic scintigraphy after the first therapies. After confirming the sufficient elevation of TSH concentration, thyroid hormone replacement was resumed one day before ^131^I administration (3.7-7.4GBq). The ablation rate of thyroid remnant at the first ^131^I therapy was evaluated by comparing ^131^I post-therapeutic images of the two treatments.

**Results:**

Three patients were administrated thyroid hormone after ^131^I therapy because of insufficient TSH concentration under thyroid hormone withdrawal. In the remaining 26 patients, 41 thyroid remnant accumulations were detected in all 26 patients at the first ^131^I therapy. Based on the second ^131^I post-therapeutic images, successful ablation was confirmed in 24 of 26 patients (92.3%) and 38 of 41 sites (92.7%), which was comparable with historically reported ablation rates.

**Conclusion:**

Thyroid hormone replacement one day before ^131^I therapy could provide a sufficiently high ablation rate in patients with DTC.

## Introduction

Radioiodine-131 (^131^I) therapy has been commonly used for well-differentiated thyroid cancer (DTC). This procedure is clinically beneficial in reducing recurrence and increasing the sensitivity of serum thyroglobulin, which reflects tumor activity of DTC (1-3).

To achieve sufficient ^131^I uptake in residual thyroid tissues and tumors, ^131^I therapy for DTC requires thyroid-stimulating hormone (TSH) elevation. Thyroid hormone replacement must be withheld for a certain amount of time in order to permit an adequate rise in TSH, ideally higher than 30mIU/L on the day of ^131^I administration. The duration of thyroid hormone withdrawal requires at least 2 weeks for triiodothyronine (T3) and 4 to 6 weeks for thyroxine (T4) (4-5). Meanwhile, thyroid hormone replacement is conventionally resumed several days after ^131^I administration. ATA guidelines and EANM guidelines recommended that thyroid hormone replacement should be resumed 2 or 3 days after ^131^I administration (1, 4). Under thyroid hormone discontinuation, most patients suffer from hypothyroid symptoms, such as fatigue, lethargy, cold intolerance, weight gain and nonpitting edema and their quality of life (QOL) is impaired. Moreover, elevated TSH may stimulate the growth of residual lesions. To palliate hypothyroid symptoms and eliminate unnecessary TSH stimulation to residual lesions, shortening the term of thyroid hormone withdrawal is desirable. If thyroid hormone replacement started earlier than usual, patients could obtain better QOL during their preparation of ^131^I therapy.

After the initiation of thyroid hormone replacement, TSH concentration gradually dissolves. It was experienced that TSH concentration was mostly higher than 30mIU/L for one or two days after the initiation of thyroid hormone replacement. For this reason, patients whose TSH levels sufficiently elevated routinely resumed thyroid hormone one day before ^131^I administration in our institution. The current study determined the efficacy of ^131^I ablation therapy with thyroid hormone replacement one day before ^131^I administration.

## Methods

### Patients

Twenty nine consecutive patients who underwent ^131^I therapies twice during 6-12 months for DTC between June 2008 and November 2010 were studied. Since all patients obviously had residual lesions by their serum thyroglobulin levels or their scintigrams at the first therapies, they underwent the second ^131^I therapies without diagnostic ^131^I scintigraphy after the first therapies. The patients comprised 10 males and 19 females, and the age range was 24 to 74 years (mean = 52.3 years). Twenty-eight were papillary carcinoma (three had the predominant follicular patterns) and one was a follicular carcinoma. All patients underwent total or near total thyroidectomy by experienced surgeons. No diagnostic ^131^I scintigraphy was performed before the first ^131^I therapy. All patients gave their informed consent for their ^131^I therapies.

### Preparation for ^131^I therapy (Figure 1)

**Figure 1:**
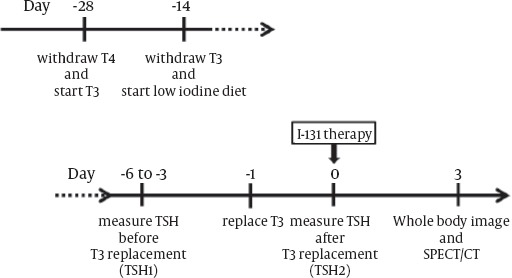
Study protocols. TSH values measured at 3-6 days before and at the day of ^131^I administration are defined as TSH1 and TSH2, respectively. T3 medications are resumed one day before ^131^I therapy. TSH, thyroid-stimulating hormone; T3, triiodothyronine; T4, thyroxine.

All patients were prepared by switching from T4 to T3 4 weeks before ^131^I therapy. T3 withdrawal and low iodine diet were started 2 weeks before ^131^I therapy. Serum TSHs were measured twice at 3-6 days before (TSH1) and at the day of ^131^I therapy (TSH2). After confirming the sufficient elevation of TSH1 concentration, T3 replacement was resumed one day before ^131^I administration. To ensure TSH2 of more than 30mIU/L, patients whose TSH1 concentrations were less than 40mIU/L were administrated T3 after ^131^I therapy.

### ^131^I therapy and post-therapeutic scintigraphy

Therapeutic doses of ^131^I were administrated. ^131^I doses were classified according to the patient’s condition: 3.7GBq for patients with lymph node metastases or without metastases, 5.55GBq for patients with lung metastases and 7.4GBq for patients with bone metastases. Post-therapeutic scintigrams were acquired 3 days after administrations, using a dual-head gamma camera equipped with high energy collimators and 3/8 inch NaI crystals, which was combined to a low-dose spiral CT in the same gantry (Symbia^®^, Siemens Medical Solutions). Whole body planar images were acquired 3 days after ^131^I therapy at scanning speed of 15 cm/min. Following planar imaging, SPECT images of the neck and chest were obtained. Additional SPECT images were acquired to cover the areas suspected of abnormal tracer accumulations in whole body planar images. SPECT data were acquired from 60 projections (20 seconds per view) with 128 × 128 matrix and reconstructed using a 3-dimensional ordered-subset expectation-maximization algorithm. As soon as SPECT data acquisition was finished, patients underwent CT transmission scans for tomography. SPECT and CT data were analyzed and co-registered using an e-soft workstation (Siemens Medical Solutions).

### Image interpretation

Two experienced nuclear medicine physicians, who were blinded to the findings of the other imaging modalities, assessed thyroid remnant accumulation at the first and second post-therapeutic scintigraphy. An ablation rate of the first ^131^I therapy was determined by comparing the scintigraphic findings of the first therapy and those of the second therapy. When their interpretation was discordant, they obtained consensus after conference.

The Student’s t-test was employed to compare the continuous variables. The Fisher exact test was used to compare the ablation rates between two groups. A p value of less than 0.05 was considered as a significant difference.

## Results

At the first ^131^I therapy, TSH1 values ranged from 16.56 to 283.30mIU/L (mean = 98.98mIU/L) in the 29 patients. T3 replacement was started in twenty-six patients with TSH1 values more than 40mIU/L (TSH1 ≧ 40mIU/L) one day before ^131^I therapy. T3 was administered to the remaining three patients with TSH1 values less than 40mIU/L (TSH1 < 40mIU/L) (16.56, 20.21, 35.16mIU/L) after ^131^I therapy.

At the second ^131^I therapy, 5 of 29 patients were administrated T3 after ^131^I therapy (3.7-5.55GBq, mean = 4.07GBq), because TSH1 concentrations were less than 40mIU/L (29.58, 31.38, 33.66, 34.36, 35.96mIU/L). T3 replacement was started in the remaining 24 patients one day before ^131^I therapy and 3.7-5.55GBq (mean = 4.32GBq) of ^131^I was administered to them.

TSH changes between TSH1 and TSH2 were analyzed. In addition, the ablation rate was evaluated in 26 patients with T3 replacement one day before the first ^131^I therapy compared to 3 patients with T3 replacement after the first ^131^I therapy.

### TSH changes between TSH1 and TSH2

Table 1 shows TSH1 and TSH2 at the first and the second therapies in all patients. The mean time periods from T3 discontinuation to TSH1 measurement in patients with TSH1 ≧ 40mIU/L (n=50) and in patients with TSH1 < 40mIU/L (n=8) were 10.2 days (8 to 11 days) and 10.4 days (10 to 11 days) respectively. There was no significant difference between the two periods.

**Table 1:**
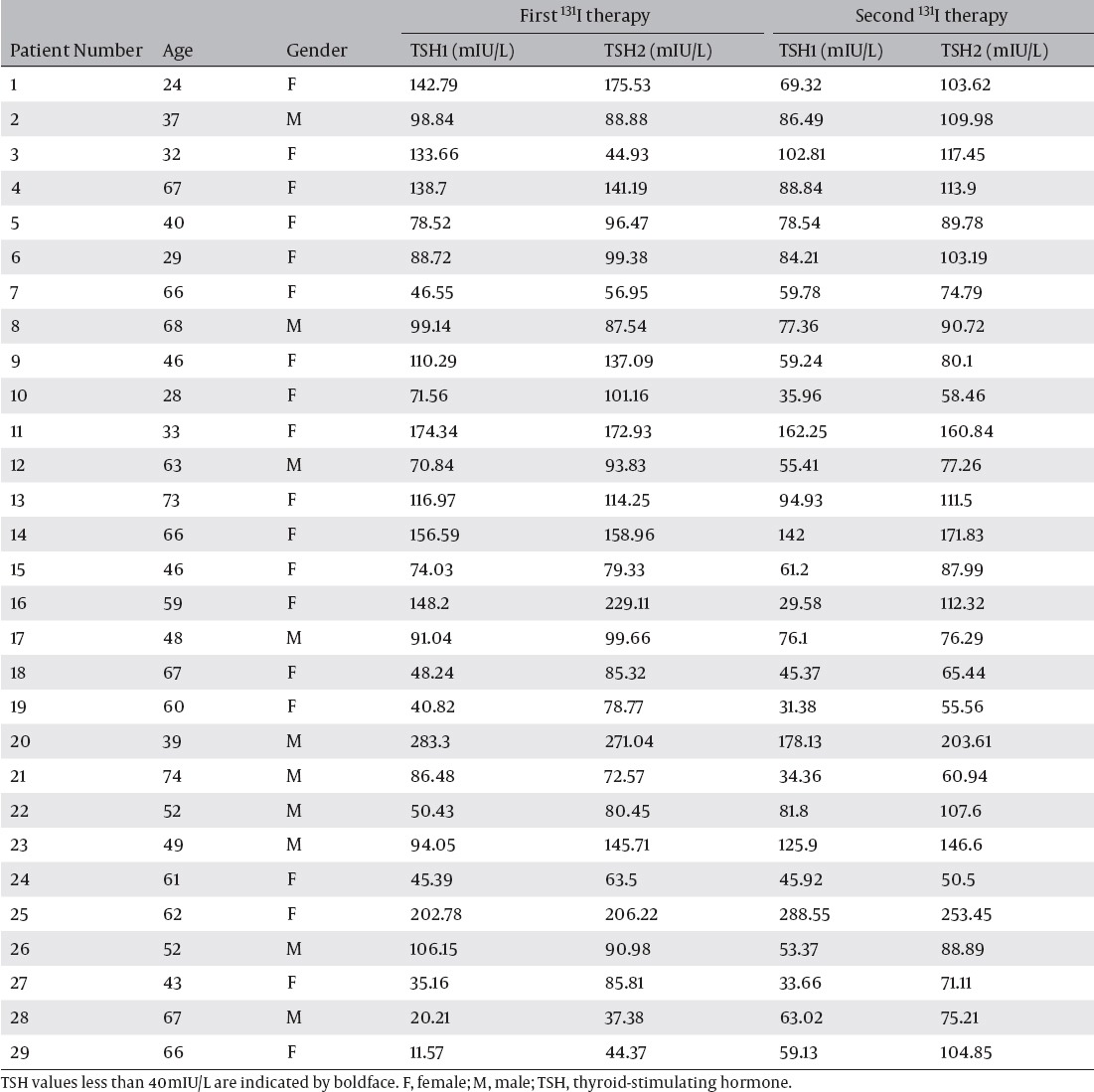
TSH1 and TSH2 values at the first and the second ^131^I therapies

**Figure 2:**
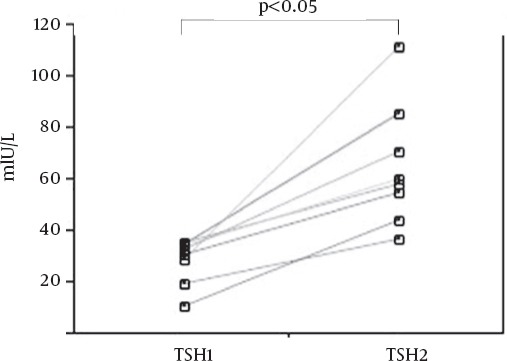
The changes between TSH1 and TSH2 in patients with TSH1 < 40mIU/L (n=8). TSH values measured at 3-4 days before and at the day of ^131^I administration represent as TSH1 and TSH2, respectively. TSH, thyroid-stimulating hormone.

**Figure 3:**
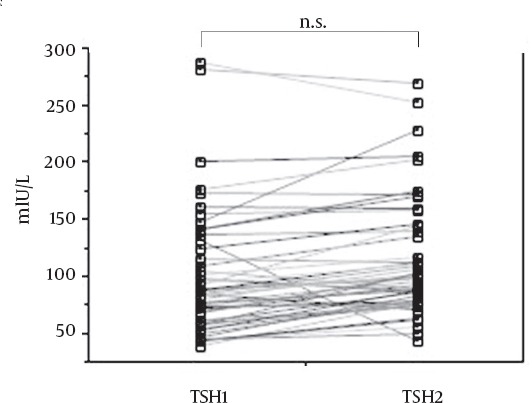
The changes between TSH1 and TSH2 in patients with TSH1 ≧ 40mIU/L (n=50). TSH values measured at 3-6 days before and at the day of ^131^I administration represent as TSH1 and TSH2, respectively. There are no patients with TSH2 < 30mIU/L. TSH, thyroid-stimulating hormone; n.s, not significant.

Figure 2 shows the changes between TSH1 and TSH2 in patients with TSH1 < 40mIU/L of the first (n = 3) and the second (n = 5) therapies. TSH2 was significantly higher than TSH1 (p < 0.05), because T3 replacement was started after measurement of TSH2 and ^131^I therapy. Figure 3 shows the changes between TSH1 and TSH2 in patients with TSH1 ≧ 40mIU/L of the first (n = 26) and the second (n = 24) therapies. TSH1 and TSH2 ranged from 40.82 to 288.55mIU/L (mean = 100.76mIU/L) and 44.93 to 271.04mIU/L (mean = 114.74mIU/L), respectively. There was no significant difference between TSH1 and TSH2 in the 50 paired TSH measurements. However, in 40 of 50 measurements, TSH values increased in spite of T3 replacement one day before measurement of TSH2 and ^131^I administration. In the remaining 10 patients, although TSH values decreased, no TSH level at the day of ^131^I therapy was less than 30mIU/L.

### Thyroid remnant accumulations in the post-therapeutic images of two treatments

**Figure 4:**
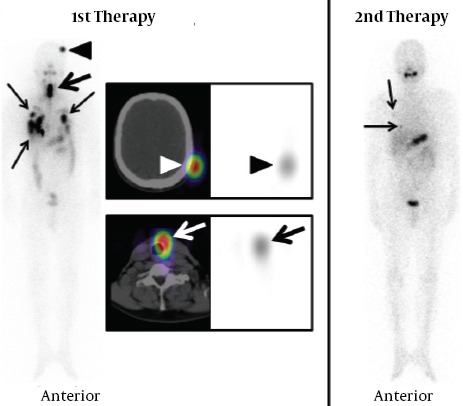
A 48-year-old male with papillary carcinoma, patient number 17 in table 1 and 2. A vertically long accumulation in the neck (wide arrow), a point-like accumulation in the left head (arrow head) and multiple accumulations in the lungs (narrow arrows) are seen with the whole body imaging obtained after the first therapy. With the SPECT/CT, the neck accumulation (wide arrows) is considered as a thyroid remnant and the left head accumulation (arrow heads) is suspected of a skin metastasis. The latter is surgically resected and proved to be a skin metastasis. The post-therapeutic scintigraphy of the second therapy detects no accumulation in the neck and can verify successful ablation. Only two faint accumulations considered as lung metastases are seen in the right lung (narrow arrows).

**Figure 5:**
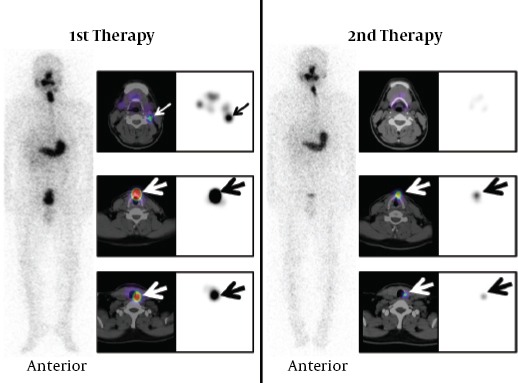
A 39-year-old male with papillary carcinoma, patient number 20 in table 1 and 2. Three accumulations are detected in the neck with the SPECT/CT obtained after the first therapy. One of them is located in the left upper neck and is suspected of a lymph node metastasis (narrow arrows). Others are considered as thyroid remnants because of their locations (wide arrows). With the SPECT/CT obtained after the second therapy, no accumulation is seen in the left upper neck. However, two accumulations considered as thyroid remnants still exist (wide arrows).

Table 2 shows the number of thyroid remnants in the first and the second ^131^I therapies in patients with T3 replacement one day before the first ^131^I therapy. Forty-one thyroid remnant accumulations were detected in all 26 patients at the first ^131^I therapies and 3 thyroid remnant accumulations were detected in 2 patients at the second ^131^I therapies. Successful ablation of the first ^131^I therapy was confirmed in 24 of 26 patients (92.3%) and 38 of 41 sites (92.7%) as judged by the second ^131^I post-therapeutic images. Figure 4 and Figure 5 show the representative scans of successful and unsuccessful ablation cases.

**Table 2:**
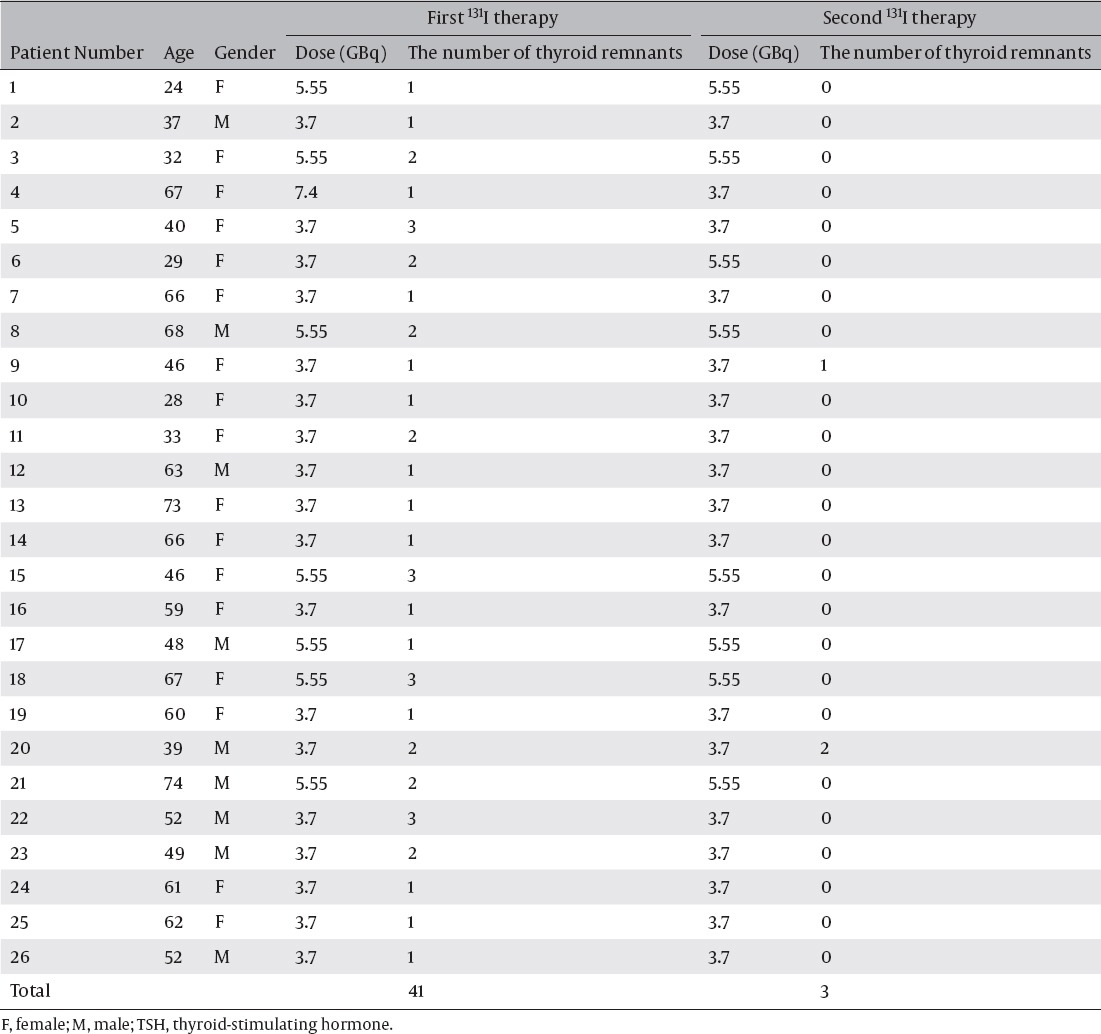
The administrated doses and the number of thyroid remnants detected at the first and the second ^131^I therapies in patients with T3 replacement one day before the first ^131^I therapy

Table 3 shows the number of thyroid remnants in the two treatments in patients with T3 replacement after the first ^131^I therapy because of TSH1 at the first therapies < 40mIU/L. Seven thyroid remnant accumulations were detected in all 3 patients at the first therapies and one thyroid remnant accumulation was detected in one patient at the second therapy. Successful ablation of the first ^131^I therapy was confirmed in 2 of 3 patients (66.7%) and 6 of 7 sites (85.7%).

**Table 3:**
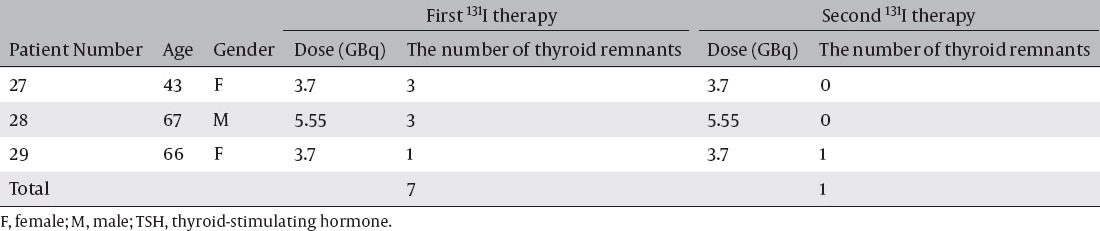
The administrated doses and the number of thyroid remnants detected at the first and the second ^131^I therapies in patients with T3 replacement after the first ^131^I therapy

Successful ablation rate was higher in patients with T3 replacement one day before ^131^I therapy than in patients with T3 replacement after ^131^I therapy. However, there was no significant difference between the ablation rates.

## Discussion

We demonstrated that T3 replacement one day before ^131^I therapy could achieve sufficient TSH concentration and a good successful ablation rate in patients with DTC. A successful ablation rate in patients with T3 replacement one day before ^131^I therapy, more than 90%, was comparable with historically reported ablation rates (6-10). The current study results indicate that T3 replacement one day before ^131^I therapy could be a clinically useful method in patients with DTC.

Therapeutic use of ^131^I is widely accepted for DTC; however, some problems arise such as decrease in patient’s QOL and stimulation to residual lesions by elevated TSH during thyroid hormone withdrawal (11-12). Recombinant human TSH (rhTSH) is available for thyroid remnant ablation and can resolve these problems (12-15). In the future, rhTSH may become the main modality of choice for ^131^I therapy. However, thyroid hormone withdrawal will be the mainstream method for a while because of insufficient evidence for rhTSH in DTC patients with metastases or recurrence. In addition, especially in developing countries, thyroid hormone withdrawal is an indispensable method because of the high price of rhTSH.

To shorten the period of the hypothyroid state and to raise TSH level, various studies have reported about thyroid hormone withdrawal, investigating, for example, its duration and timing, using at times T4 only, and at other times using T3 and T4 together (16-20). In one study (19), TSH values were repeatedly measured after total thyroidectomy or after withdrawal of suppressive T4 therapy in preparation for ^131^I therapy. The time required for TSH levels to reach more than 30mIU/L was 8-26 days after thyroidectomy and 9-29 days after T4 withdrawal. Another study (18) investigated the TSH concentration with a conventional, widely used regimen of substitution of T3 for T4, 6 weeks prior to ^131^I therapy and then its subsequent discontinuation 2 weeks prior to therapy. In this report, 6 (11.5%) of 52 patients did not achieve a TSH level of 30mIU/L two weeks after T3 withdrawal. These reports indicated the difficulty of changing the initiation time of thyroid hormone withdrawal because of wide inter-individual variation in TSH concentration.

In this study, the time periods between discontinuing of T3 and TSH1 measurement were 8 to 11 days (mean = 10.2 days). Although the period of T3 discontinuation was short, 93.1% of the patients in the first therapy and 96.6% of the patients in the second therapy had TSH1 ≧ 30mIU/L under normal low iodine diet (Table 1). These results indicated that hypothyroid state during thyroid hormone withdrawal could be shortened further. Further study is needed in this subject.

As for the initiation time of thyroid hormone replacement, ATA guidelines and EANM guidelines stated that thyroid hormone replacement should be resumed 2 or 3 days after ^131^I therapy for DTC (1, 4). The current study demonstrated that the initiation time of T3 replacement could be moved forward several days compared with the conventional method. T3 replacement one day before ^131^I therapy would shorten the period of the hypothyroid state and decrease unnecessary TSH stimulation to residual lesions during patients’ preparation for ^131^I therapy. Considering the difference of pharmacokinetics between T4 and T3, the initiation time of T4 replacement might be earlier than that of T3.

In the current study, to ensure that TSH values at the day of ^131^I administration (TSH2) be more than 30mIU/L, the TSH changes analysis was excluded and T3 was started after ^131^I therapy in 3 patients at the first therapy and 5 patients at the second therapy whose TSH values at 3-6 days before the first ^131^I therapy (TSH1) were less than 40mIU/L. In the 50 patients whose TSH1 values were more than 40mIU/L, 10 (20%) patients had decrease in TSH2 values because of T3 replacement one day before ^131^I therapy. However, TSH2 values did not decrease to less than 30mIU/L in any patient on the day of ^131^I therapy. These results would validate the exclusion criterion in this study. However, the exclusion criterion should be further evaluated in a large cohort.

An issue may arise as to whether thyroid hormone interrupts ^131^I uptake in thyroid remnants and residual tissues, since thyroid hormone transmutes into iodine. However, many reports on the utility of rhTSH under thyroid hormone continuation for ^131^I therapy suggest that early thyroid hormone replacement would not interfere with ^131^I accumulation (14-15).

There were some limitations in the current study. The study was a retrospective study with small population. It did not evaluate patients’ symptoms and QOL after ^131^I therapy. To resolve these problems, further studies are needed.

## Conclusion

Thyroid hormone replacement one day before ^131^I therapy could provide a sufficiently high ablation rate in patients with DTC. Compared with thyroid hormone replacement several days after ^131^I therapy, this alternative method would be beneficial in shortening hypothyroid periods and eliminating unnecessary TSH stimulation to residual lesions.

## Conflicts of interest

There are no conflicts of interest.
